# Synopsis of 30 Cases of Ovariotomy, Occurring in the Practice of the Author

**Published:** 1855-05

**Authors:** Washington L. Atlee

**Affiliations:** Philadelphia


*Synopsis of* 30 *cases of Ovariotomy, occurring in the practice of the author.* By WASHINGTON L. ATLEE, M.D., of Philadelphia. (American Journal of Medical Sciences, April, 1855.)—It is impossible to arrive at the true results of any operation—even the bare proportion of its death and recoveries—however large our statistics may be, unless we have all the necessary data before us. This is peculiarly the case with ovariotomy. From the general odium attached to it, there can be little doubt that there have occurred many fatal cases of its practice, which have never been given to the profession. The published results must, consequently, be viewed as only an approximation to the true proportion. Dr. Atlee's synopsis is peculiarly valuable for being free from this objection. The number of his cases is considerable; the operation was performed in every instance by the same person; one, too, whose large experience upon every point connected with the subject, insured the greatest probabilities of success, and the results are faithfully given us. That the profession may see at a glance what these results are, we shall give them a short analysis of the "synopsis."

We shall take the liberty, in the first place, of withdrawing four of the cases from the record, for the sufficient reason that in none of them was the operation completed. The tumor was left undisturbed in every instance, an exploratory incision alone being made. Of these four cases, three were uterine and non-adherent, and one extra-uterine and firmly adherent. Recovery from the abdominal section took place in all.—Death from erysipelas occurred six months after operation in one case. One died from the progress of the disease, between three and four years afterwards, and two are still alive, one of who was operated on five, and one nearly four and a half years ago. "Improved health since the operation" is stated to have taken place in one of these cases. In the other, "although the tumor was not removed, the operation was considered to have warded off impending death."

Of the twenty-six remaining cases, which we presume are all in which Dr. A. has operated, twenty-two were ovarian, and four extra-uterine tumors. Of the ovarian cases, eleven deaths are recorded and eleven recoveries. Of the extra-uterine, two deaths and two recoveries.

Of the thirteen recoveries, ten are now alive—five of whom were operated on between March, 1814, and January 1852—one in September 1853, and four between April and December, 1854—and three are dead, one dying thirty-nine days after the operation of cholera morbus, from eating heartily of duck, &c.—one thirty days after, of starvation, caused by excessive irritability of stomach, the consequence of pregnancy. (Dr. Atlee queries whether the production of abortion, the patient being two months gone, might not have preserved life in this case;) and one from phthisis pulmonalis three years after the operation.

The condition of health of the ten now living, a most important consideration, is not noted, excepting in one instance, where it is represented as being perfect. Two cases have been pregnant but miscarried, and one has given birth to two large, healthy children, after being operated on.

In the thirteen which died, death was caused by peritonitis in four cases, by exhaustion in five, by hemorrhage in one, secondary hemorrhage in two, and by grangrenous perforation of the jejunum in one.

The period of death, after operation, was nine hours in one case; thirteen hours in one case; on the third day in five cases; on the fifth day in two cases; on the sixth, three cases; and on the twenty-second day one case.

The average time of death of these cases was about five days. The operation was performed with the hope of arresting impending death in seven cases, four of which terminated fatally.

To sum up, the operation was unsuccessful, from not being completed in four cases.

Of the twenty-six remaining cases, in all of which extirpation was performed, 15 or 57 per cent. of the whole number died within forty days.

Of the eleven remaining cases, one died of phthisis pulmonalis, an hereditary disease, three years after operation, and ten were alive up to the period of publication of Dr. Atlee's paper.

The condition of health of those still living is, as before stated, not noted, excepting in one instance.

We have no doubt that these results are, for reasons before stated, far more correct and reliable than any which have been previously published. They conclusively show that the very strong prejudice entertained by a large part of the profession against ovariotomy, is well-founded and proper. Nor is it an easy matter to justify it on other grounds. Few will admit that there exists the same urgent necessity for it that there does for strangulated hernia, or amputation, or ligature of large arteries, or lithotomy. All of these operations are unavoidable. The case is entirely different with ovariotomy. Women, frequently live even to old age, with ovarian disease, and many cases are recorded, in which the effused fluid has spontaneously disappeared. Upon the ultimate fate of the *recoveries*, too, we are in complete ignorance. Upon this point Dr. Ashwell observes:

"In these remarks, (alluding to a statement of Mr. Safford Lee,) I certainly concur, especially as to the ultimate, and in most instances early mortality of many of the patients said to have recovered. My own inquiries have furnished me with some painful knowledge of this kind." (*Treatise on the Diseases Peculiar to Women.*)

With these facts before us, we think it is the bounden duty of the profession to discountenance ovariotomy. One of the most frightful operations in surgery, its want of success, even when performed by an expert, will, we trust, add still further to the odium which has, heretofore, attached to it.

